# Transcriptomic analysis of Crandell-Rees feline kidney cell infections with field and vaccine feline calicivirus strains

**DOI:** 10.1016/j.virusres.2025.199681

**Published:** 2025-12-21

**Authors:** Emily Kwan, Alistair R. Legione, Carol A. Hartley, Joanne M. Devlin

**Affiliations:** Asia-Pacific Centre for Animal Health, Melbourne Veterinary School, Faculty of Science, The University of Melbourne, Building 400, Parkville, 3010, Victoria, Australia

**Keywords:** Transcriptomic, Feline upper respiratory tract disease, Host response, Viral pathogenesis

## Abstract

Feline upper respiratory tract disease (URTD) is a significant health concern in crowded environments, such as catteries and shelters. *Feline calicivirus* (FCV), which is endemic in domestic cats, is a major contributor to URTD and can cause a range of diseases that vary in severity. Unlike many other caliciviruses, including human caliciviruses and noroviruses, FCV replicates efficiently in cell culture and is a well-established model for studying calicivirus-host cell interactions. In this study, RNA-sequencing was used to characterise host and viral transcription profiles in Crandell-Rees feline kidney (CRFK) cells infected with either the attenuated F9 vaccine strain or a virulent field strain associated with virulent systemic FCV (VS-FCV) disease. At six hours post-infection, both strains induced the upregulation of inflammatory and stress response pathways, while genes involved in metabolism, cell signalling, and extracellular matrix maintenance were downregulated. Between field and vaccine strains, pathways related to membrane signalling and cytoskeletal organisation were uniquely altered in the field strain, while the F9 vaccine strain distinctly altered chromatin organisation, cytokine signalling and mitochondrial metabolism. These findings demonstrate the virulence of FCV strains differentially influences host gene expression in CRFK cells, which may inform host-pathogen interactions that contribute to FCV pathogenesis and variations in disease outcomes.

## Highlights

• FCV infections in CRFK cells show the effects of strain virulence on host responses.
• Field and vaccine strains upregulated inflammatory genes, such as TNF-α and SELE.
• PI3K pathway inhibitors were downregulated during FCV infection.
• Interleukin and histone genes were differentially expressed between FCV strains.
• CRFK cells are relevant *in vitro* models to study FCV pathogenesis and host response.

## 1 Introduction

*Feline calicivirus* (FCV) is a highly contagious viral pathogen that causes upper respiratory tract disease (URTD) in cats*.* Most FCV infections are acute, and cause clinical signs such as rhinosinusitis, conjunctivitis, and ulcerations on the eyes, tongue, gums, and oral mucosa (Hofmann-Lehmann et al., 2022). Some cats can develop chronic or persistent infections following the acute phase and may continue to shed the virus without clinical signs (Coyne et al., 2006). Infections with virulent systemic-FCV (VS-FCV) strains are associated with extensive vascular and epithelial injury, oedema in multiple limbs, and diseases such as pneumonia and chronic gingivostomatitis (Atkinson, 2008; Bordicchia et al., 2021; Monne Rodriguez et al., 2014). While disease severity is likely associated with viral strain virulence, host responses can influence viral pathogenesis and disease susceptibility (Nathanson and González-Scarano, 2016). Therefore, characterising both host and viral gene expression profiles during FCV infections with strains that differ in virulence may show host-pathogen interactions that contribute to variations in disease outcome.

Caliciviruses are non-enveloped virions which contain a positive-sense single strand RNA genome enclosed within an icosahedral capsid. The viral structural proteins VP1 and VP2, which form the capsid, bind to the feline junctional adhesion molecule-A (fJAM-A) located at tight junctions of endothelial and epithelial cells, as well as on the surface of leukocytes and platelets (Lu et al., 2018; Pesavento et al., 2011). The major capsid protein VP1 facilitates viral entry through interactions with cellular endocytosis mechanisms, which causes endosomal acidification following viral internalisation. This acidification enables the uncoating of the viral capsid and release of viral RNA into the cytoplasm (Stuart and Brown, 2006). The viral protein genome (VPg) covalently linked to the 5′ end of the RNA interacts with host translation initiation factors to initiate genome replication. Non-structural (NS) proteins translated from open reading frame (ORF) 1 of the genomic RNA utilise host cellular machinery to form replication complexes (RC), which protect the viral genome from host immune responses during replication (Penaflor-Tellez et al., 2019). The subgenomic RNA, post-translationally transcribed by the RNA-dependent RNA polymerase (NS7), contains ORF2 and ORF3, which encodes structural proteins required for capsid assembly. Matured virions are transported to the cytoplasm and released through apoptosis (Al-Molawi et al., 2003; Natoni et al., 2006; Roberts et al., 2003; Sosnovtsev et al., 2003).

FCV is a member of the family *Caliciviridae* and belongs to the genus *Vesivirus*, whereas human caliciviruses are classified within the genus *Norovirus* or *Sapovirus* (Burrell et al., 2017). Challenges with growing human caliciviruses in cell culture have led to the use of other caliciviruses such as FCV in cats and murine norovirus (MNV) in mice to investigate host-pathogen interactions and cellular responses contributing to calicivirus pathogenesi*s* (Cheng et al., 2022; Ohmine et al., 2018). By studying the host and virus transcriptomes profiles during FCV infection, we may gain a better understanding of host and viral factors that contribute to viral pathogenesis and variation in disease outcome. This study characterised the transcriptomes of Crandell-Rees feline kidney (CRFK) cells infected with field or vaccine FCV strains to better understand cellular responses to FCV strains with different virulence characteristics.

## 2 Materials and methods

### 2.1 Viruses and cells

Archived isolates of FCV field strains, collected from cats with URTD and confirmed positive using qPCR, were used to infect monolayers of CRFK cells (Table 1) (Crandell et al., 1973; Stockley, 2013). The CRFK cell line, derived from the kidney of a clinically healthy 12-week-old female cat, is commercially available from the American Type Culture Collection (ATCC CCL-94) (Vaz et al., 2016). The F9 vaccine strain, originally isolated from a commercial F9 vaccine and plaque purified on CRFK cells, was included for comparison (Bittie and Rubic, 1976; McDonagh et al., 2015). Cell monolayers were grown in growth media containing Dulbecco’s Modified Eagle basal media (DMEM, Sigma), 5 % v/v foetal bovine serum (FBS, Sigma), 10 mM N-2-hydroxyethylpiperazine-N’−2-ethanesulfonic acid (HEPES) (pH 7.7) and antimicrobials (50 μg/mL ampicillin, 5 μg/mL amphotericin B). Cells were incubated at 37 °C in a humidified atmosphere of 5 % v/v CO_2_ and passaged (1:5) every three to four days using 5 % Trypsin/Versene.

**Table 1:** Field and vaccine FCV isolates used in this study.

Virus ID	Strain	Year of isolation	Disease	Site
86/68 (Sykes et al., 1998)	Field	1968	Mucopurulent discharge	Conjunctiva
F9 (Bittie and Rubic, 1976)	Vaccine	1992	N/A^3^	N/A^3^
3197 (Stockley, 2013)	Field	2012	VSD^1^	Nasal
3198 (Stockley, 2013)	Field	2012	Oral ulcerations and URTD^2^	Nasal
3199 (Stockley, 2013)	Field	2012	URTD^2^	Nasal
3200 (Stockley, 2013)	Field	2012	Oral ulcerations and swollen limbs	Nasal
3201 (Stockley, 2013)	Field	2012	Oral ulcerations and URTD^2^	Nasal
14 (Symes et al., 2015)	Field	2014	URTD^2^	Oral

1 VSD= Virulent- systemic disease.

2 URTD= Upper respiratory tract disease.

3 N/*A*= Not available.

### 2.2 One-step viral growth curves

Cell monolayers of CRFK cells were cultured in six-well plates and inoculated at a multiplicity of infection (M.O.I.) of 5 median tissue culture infective dose (TCID_50_) per cell, in triplicate. Uninfected cell monolayers were inoculated with growth media only. After 1 hour of viral adsorption, cells were washed twice with phosphate-buffered saline (PBS, pH 7.4, 137 mM NaCl, 8.2 mM Na_2_HPO_4_, 2.7 mM KCl) and maintenance media containing DMEM with 1 % v/v FBS, 10 mM HEPES pH 7.7 buffer solution and antimicrobials (50 μg/mL ampicillin, 5 μg/mL amphotericin B) was added to each well. At six timepoints, 1-, 3-, 9-, 12-, 18- and 24-hours post-infection (h.p.i), cell monolayers were examined, and both the cells and supernatant were collected to measure total virus. Samples were subjected to one freeze-thaw cycle (frozen at –80 °C and thawed at room temperature) to release intracellular virus. Viral titres of each FCV strain were determined across the six timepoints using TCID_50_ titration assays (Smither et al., 2013). Mean titres from triplicate inoculations were compared between strains at each timepoint using Student’s *t*-test, with P values < 0.05 considered significant. The 6 h.p.i selected for transcriptomic analysis coincided with the onset of exponential viral replication and early phase of infection.

### 2.3 Sample preparation for transcriptomic study

Cell monolayers of CRFK cells were cultured in 6-well plates and inoculated using the field strain 3197 or the F9 vaccine strain at an M.O.I. of 5 TCID_50_ per cell. At 6 h.p.i, the supernatant of each triplicate was collected in 1 mL aliquots and stored at –80 °C. The remaining material of each well was scraped into the existing media and centrifuged at 300 × *g* for 5 min. Cell pellets were resuspended in RLT Plus buffer (RNeasy Plus Mini Kit, Qiagen) with 1 % v/v β-mercaptoethanol and stored at –80 °C. Total RNA was extracted using the RNeasy Plus mini kit (Qiagen), according to the manufacturer’s instructions, and quantified using the Qubit RNA High Sensitivity Assay (Thermofisher). RNA quality was assessed using the Agilent 4200 Tapestation system (Agilent Technologies, Santa Clara, *CA*). All samples used for Illumina RNA-sequencing showed RNA integrity numbers (RIN) > 8.

### 2.4 Illumina RNA-sequencing

The preparation of cDNA libraries and RNA-sequencing were performed at the Australian Genome Research Facility (AGRF, Melbourne). Briefly, the TruSeq stranded mRNA library preparation kit (Illumina Inc., San Diego) was used to construct cDNA libraries and samples with the correct fragment size (∼260 bp) were confirmed using the Agilent 2200 Tapestation. Libraries were sequenced at a depth of 20 M on the NextSeq 500 sequencing platform (Illumina Inc., San Diego) to produce 150 paired-end reads.

### 2.5 Quality control and data processing

Raw RNA-sequencing data was uploaded to the Galaxy web platform and analysed on the public server *usegalaxy.org.* Read quality was assessed using FastQC version 0.11.8 (Afgan et al., 2016; Blankenberg et al., 2014), and low-quality reads and Illumina adapter sequence were trimmed using CutAdapt (Jalili et al., 2020; Martin, 2011). The remaining reads were mapped to the annotated Fca126 domestic cat genome (Lopez et al., 1996) (general feature format and FASTA format), retrieved from the NCBI database (RefSeq accession no. GCF_018350175.1), and the corresponding viral genomes of the field strain 3197 (Stockley, 2013) and FCV F9 vaccine strain (GenBank Accession No. M86379.1) (Carter et al., 1992) using RNAstar (Widmann et al., 2012). Host and viral transcription was measured using featureCounts (Liao et al., 2013), and uploaded to the interactive-RNA-seq platform Degust for differential analysis using Voom/Limma version 4.2 (Law et al., 2014; Powell, 2019; Ritchie et al., 2015).

### 2.6 Differential gene expression analysis

Differentially expressed host and viral genes were filtered and analysed in the RStudio environment version 2023.06.1 + 524. To account for variations between field and vaccine infection samples, the counts per gene across the cat genome were normalised to log_2_ counts per million (CPM) for each infection or control group. Using Geneious Prime 2023.0, viral reads across the FCV genome in the field and vaccine infection groups were visualised and the counts per ORF were normalised to log_2_ CPM. Host and viral genes or gene regions with at least 10 CPM mapped reads in at least one treatment group, a log_2_ fold change (LFC) greater than 2 or less than −2, and a false discovery rate (FDR) less than 0.05 were considered significantly differentially expressed. Host genes that passed these filters were assigned either up- or downregulated and are described in this study as differentially expressed host genes compared to the uninfected samples. Viral ORFs that met these conditions were considered significantly differentially expressed in the vaccine strain compared to the field strain.

### 2.7 Enrichment of host genes with gene ontology

Pathway analysis was performed using the online PANTHER database version 17.0. Gene ontology (GO) terms were retrieved from the Uniprot database (Thomas et al., 2022) and biological processes associated with the differentially expressed host genes were analysed for pathway enrichment (Mi and Thomas, 2009). Statistical enrichment analysis categorised enriched GO terms into three functional groups: molecular functions, biological processes, and cellular components, with P-values < 0.05 considered significant (Mi et al., 2019).

## 3 Results

### 3.1 Variations between FCV field and vaccine strains

**Fig. 1:**
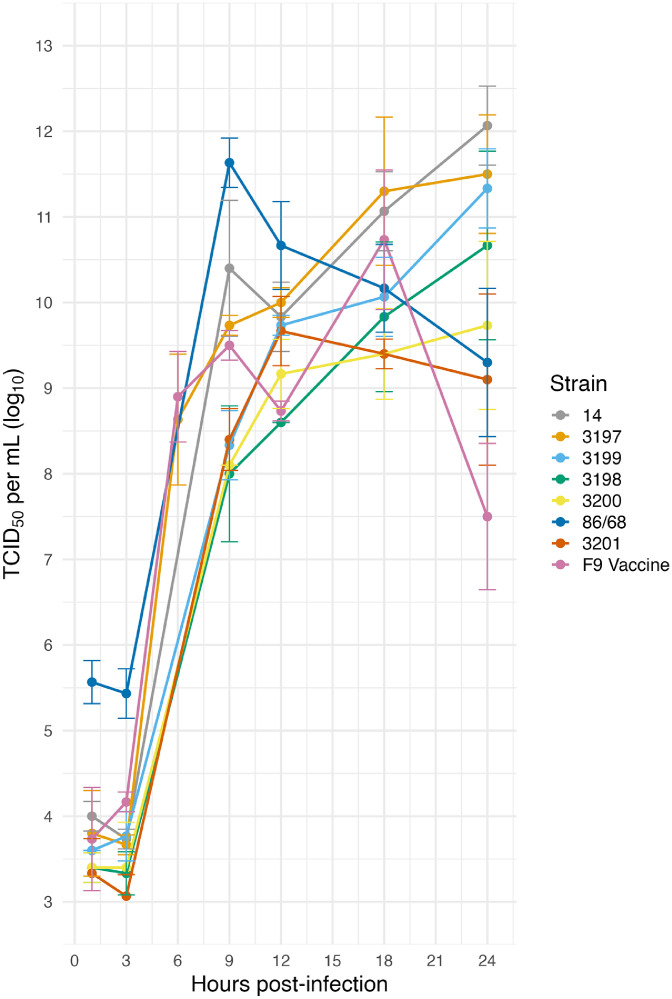
One-step growth curves of FCV field and vaccine strains in CRFK cells infected at an M.O.I. of 5 TCID_50_ per cell. Viral titres of the supernatant from triplicate wells at the indicated timepoints were determined by TCID_50_ assays. The mean TCID_50_ titre is shown with the error bars representing the standard deviations at each timepoint.

Full-genome sequencing of FCV field strains 3197 and 3198 showed a high sequence identify of 98.8 % (Stockley, 2013). Alignment using MAFFT (Katoh and Standley, 2013), showed that field strain 3197 shared 79.4 % nucleotide identity with the F9 vaccine strain. Within ORF2, the nucleotide sequence identity among field strains 3197, 3200 and 3201, was approximately 80 % (Stockley, 2013). Infected CRFK cell monolayers developed visible cytopathic effects (CPE) from 3 h.p.i. Despite infections at a high M.O.I, residual infectious virus was detectable at 18 and 24 h.p.i, which likely represents the accumulation of virus released earlier in infection, rather than on-going viral replication. Infected cells appeared shrunken, rounded and clustered around large areas of cell lysis (Kim et al., 2010; Sosnovtsev et al., 2003; Yang et al., 2020), while uninfected control monolayers showed no CPE over the 24 hour period. One-step growth curves indicated replication kinetics among FCV field and vaccine strains are similar over 24 h, with the F9 vaccine strain showing the lowest viral titre among strains at 24 h.p.i. (Fig. 1). Statistical analysis using Student’s T-test showed significant differences in viral titres among field strains, and between several field strains and the vaccine strain at each timepoint. However, not all comparisons were statistically significant (Supplementary Table 1).

### 3.2 Host and viral RNA are distinguishable in FCV in vitro infections

**Fig. 2:**
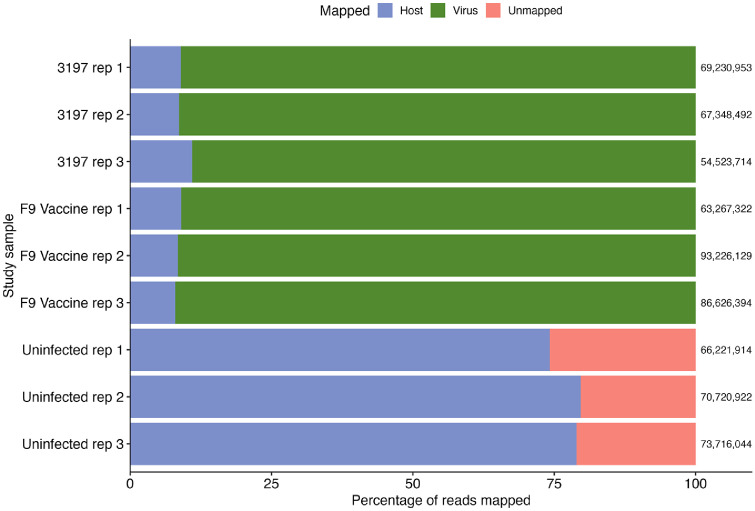
Percentage of the total reads in the FCV infected and uninfected groups in triplicate (rep) that mapped to the domestic cat genome Fca126 (Genbank acc. GCA_018350175.1) and the respective FCV field and vaccine strains. The total number of reads of each sample is shown at the right end of each sample. The unmapped reads are also shown.

**Fig. 3:**
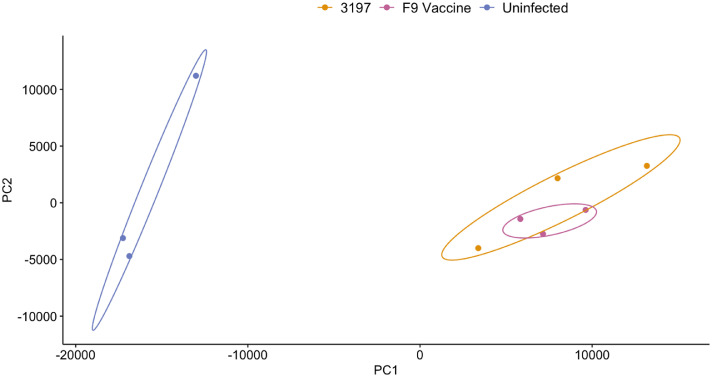
Principal component analysis (PCA) plot of the host transcription profiles in CRFK cells infected with the field strain 3197 (yellow), F9 vaccine strain (pink) and the uninfected samples (purple). The data points show biological replicates of each group with 95 % confidence ellipses.

Transcriptomes of FCV-infected and uninfected CRFK cells were collected six h.p.i and show host and viral reads during early stages of viral infection. Approximately 75 % of high-quality reads from uninfected samples mapped to the cat genome, while the remaining reads did not align to either the cat genome, the FCV 3197 field strain or F9 vaccine strain genomes. In samples infected with FCV, over 85 % of reads mapped to the respective viral genomes and less than 15 % of reads aligned to the cat genome (Fig. 2). The presence of both host and viral reads in FCV-infected samples confirmed successful viral infection and indicates comparable viral loads between the field and vaccine infection groups. Principal component analysis (PCA) of normalised host reads showed distinct clusters of infected and uninfected samples, with field and vaccine FCV infection groups clustering closely together (Fig. 3). The separation along PC1 corresponded to infection status, which indicates the variation in host gene expression is due to FCV infection rather than strain-specific differences.

### 3.3 Field and vaccine strains induce differential host gene expression compared to uninfected cells and show similar viral transcription profiles

**Fig. 4:**
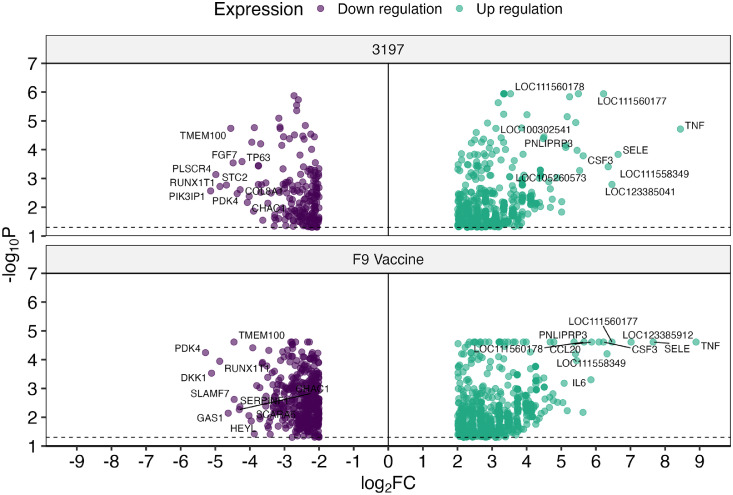
Volcano plots of the total differentially expressed host genes in CRFK cells infected with FCV field strain 3197 or the F9 vaccine strain. Host gene expression in samples with FCV infections was compared to the uninfected cells and the significantly up- and downregulated genes are shown with the top 10 up- and downregulated genes labelled.

**Fig. 5:**
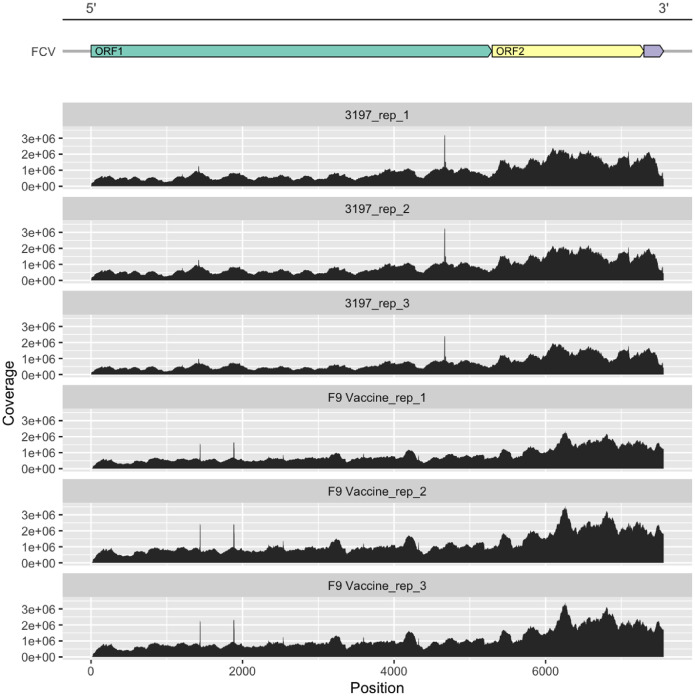
Coverage plots of viral reads in FCV infection groups that mapped to the respective field strain 3197 (top three panels) and F9 vaccine strain (bottom three panels). Coverage plots show the depth of coverage (Y-axis) at each nucleotide position (X-axis). The schematic diagram of the organisation of ORFs in FCV is shown.

Host gene expression analysis of FCV field and vaccine infection groups showed over 17,000 significantly differentially expressed genes compared to uninfected samples (Fig. 4). In cells infected with the field strain 3197, a total of 17,437 host genes were differentially expressed, of which 17,247 were upregulated and 190 were downregulated. In samples infected with the F9 vaccine strain, 17,369 host genes were differentially expressed. Of these, 16,965 were upregulated and 404 were downregulated. Differential viral ORF expression analysis between field and vaccine strains did not show significant differences in viral mRNA abundance between infection groups (Fig. 5). However, viral read coverage across ORF1 showed minor variations in abundance between field and vaccine strains. Specifically, a prominent peak of ten nucleotides spanning between nucleotide position 4633 to 4672 was observed in the field strain, while two prominent peaks between nucleotide positions 1436 to 1444, and 1880 to 1893 were identified in the vaccine strain.

### 3.4 Similarities in differentially expressed host genes in field and vaccine FCV infections

**Fig. 6:**
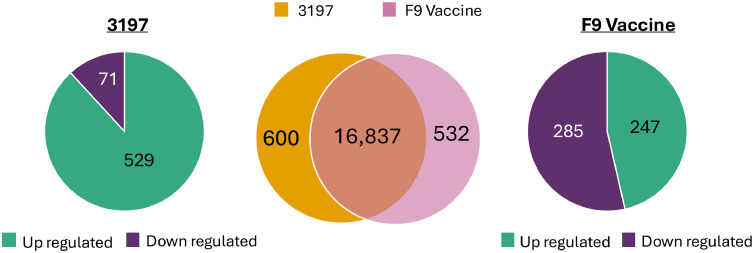
The number of differentially expressed host genes common to both field and vaccine FCV infection groups and unique to each infection group is shown by the middle circles (pink and orange). The total number of up- and downregulated genes unique to each infection group is shown for the 3197 field strain (left) and the F9 vaccine strain (right).

Comparison of differentially expressed host genes between field and vaccine infection groups showed 16,837 genes commonly dysregulated across both groups (Fig. 6). Among these, 16,718 were upregulated and 119 were downregulated. Pro-inflammatory cytokines and chemokines, including TNF-α, CSF3 and CCL4, were upregulated in both infection groups, alongside genes involved in immune cell adhesion and inflammatory responses (SELE), as well as stress response mediators such as HSP70 and PNLIPRP3. Conversely, genes associated with PI3K/AKT-mTOR signalling, cell survival and homeostasis were downregulated in both FCV infection groups compared to uninfected cells. This included decreased transcription of pro-survival and metabolic regulators (PI3K, FGF7, PDK4, TP63 and STC2), and genes involved in maintaining the integrity of the extracellular matrix (COL8A1), oxidative stress responses (CHAC1) and transcription regulation (RUNX1T1). The ten most strongly up- and downregulated genes in both FCV infection groups are shown in Table 2.

**Table 2:** The top 10 up- and downregulated genes in both field (3197) and F9 vaccine infected CRFK cells. Gene expression levels are represented by log_2_ fold change (LFC) and are presented in order of expression in field infection samples. The false discovery rate (FDR) for each gene was < 0.01.

Upregulated	Gene ID	Gene function	3197	F9 vaccine
			LFC^1^	LFC^1^
	TNF- α	Cytokine	8.44	8.90
	SELE	Cell adhesion	6.64	7.66
	LOC123385041	N/A^2^	6.46	5.63
	LOC111558349	N/A^2^	6.36	6.32
	LOC111560177	Growth-regulated α	6.22	6.47
	CSF3	Cytokine	5.63	6.22
	HSP70	Stress regulator	5.53	5.44
	LOC111560178	Growth-regulated α	5.50	5.66
	PNLIPRP3	Metabolism	5.46	6.09
	Chemokine ligand 4	Chemokine	5.42	4.77
Down regulated	PIK3IP1	PI3K inhibitor	−5.14	−3.43
	PLSCR4	Transmembrane protein	−4.98	−2.93
	RUNX1T1	Transcription regulator	−4.87	−4.87
	STC2	Glycoprotein hormone	−4.68	−3.51
	TMEM100	Transmembrane protein	−4.55	−4.46
	FGF7	Growth factor	−4.49	−3.17
	PDK4	Metabolism	−4.37	−5.28
	COL8A1	Collagen protein	−4.28	−3.65
	TP63	Transcription factor	−4.23	−2.89
	CHAC1	Metabolism	−4.07	−4.31

1 P-values < 0.05 with LFC > 2 for upregulated or < −2 for downregulated genes were considered significant.

2 N/*A*= Not available.

### 3.5 Differences in differentially expressed host genes between field and vaccine FCV infections

Between field and vaccine infection groups, 600 host genes were uniquely differentially expressed in cells infected with the field strain. In the vaccine infection group, 532 host genes were uniquely differentially expressed. The top ten up- and downregulated genes unique to each infection group is shown in Table 3. In cells infected with the field strain, genes involved in cell-signalling and membrane remodelling were uniquely upregulated compared to the vaccine strain. Conversely, the uniquely downregulated genes in the field infection group were involved in vesicle exocytosis cell development and metabolic function. In samples infected with the vaccine strain, the uniquely upregulated genes were associated with chromatin organisation (H4C16) and transcription activity, alongside genes regulating the activation of intracellular signalling cascades (TC2N and GNAT1) and innate immune signalling (IL22RA1). The distinct downregulated genes in the vaccine infection group showed a reduction in gene expressions involved in growth-factor signalling and cellular stress mechanisms.

**Table 3:** Comparison of differentially expressed host genes between field (3197) and vaccine (F9) infection of FCV in CRFK cells, compared to uninfected cells. The top 10 up- and down-regulated genes are shown with the gene expression levels represented by log_2_ fold change (LFC).

Upregulated	Gene ID	Gene function	3197		Gene ID	Gene function	F9 vaccine	
			FDR^1^	LFC^2^			FDR^1^	LFC^2^
	GPR137C	G Protein-coupled receptor	<0.01	4.14	H4C16	Histone	0.01	4.66
	LOC109497821	N/A^3^	<0.01	3.88	TC2N	Cell regulator	<0.01	4.26
	KCNJ8	Cell regulator	<0.01	3.86	GNAT1	Signal transductor	0.01	4.11
	PTGFR	G Protein-coupled receptor	0.04	3.80	LOC123382421	N/A	0.01	4.06
	LOC109496048	N/A^3^	0.04	3.73	LOC111557979	N/A	0.01	4.00
	LOC123380461	N/A^3^	0.01	3.71	LOC109501928	N/A	0.03	3.85
	LOC123381473	N/A^3^	0.03	3.70	LOC123385318	N/A	0.02	3.73
	LOC123386400	N/A^3^	0.02	3.63	LOC109500880	N/A	<0.01	3.62
	LOC109500879	N/A^3^	<0.01	3.62	IL22RA1	Interleukin	0.04	3.59
	USH1C	Cell development	0.04	3.51	LOC109497418	N/A	0.03	3.57
Down regulated	DOC2B	Exocytosis	0.02	−3.28	TRIB3	Cell metabolism	0.04	−3.87
	CA2H3orf62	N/A^3^	0.03	−3.25	DYRK3	Kinase enzyme	0.01	−3.71
	LOC123380014	N/A^3^	<0.01	−3.13	TMEM45B	Transmembrane protein	<0.01	−3.64
	IGFBP5	Growth factor	0.01	−3.05	CCN4	Growth factor	0.02	−3.46
	LIX1	Cell development	<0.01	−3.04	DDX28	RNA regulator	0.01	−3.40
	SLIT2	Cell regulator	<0.01	−3.03	TRMT10C	tRNA modification	0.01	−3.32
	STAP1	Cell regulator	<0.01	−2.98	GHR	Growth hormone	0.01	−3.31
	RLBP1	Cell metabolism	0.02	−2.96	BTBD8	N/A	<0.01	−3.29
	ASPA	Cell metabolism	0.02	−2.87	TRIM13	Immune regulator	<0.01	−3.29
	LOC123380366	N/A^3^	<0.01	−2.86	LOC111560210	Chromatin remodelling	<0.01	−3.27

1 FDR = False discovery rate.

2 P-values < 0.05 with LFC > 2 for upregulated genes or < −2 for downregulated genes were considered significant.

3 N/*A* = Not available.

### 3.6 Differentially expressed host genes in field and vaccine infection groups differentially regulate cellular pathways

**Fig. 7:**
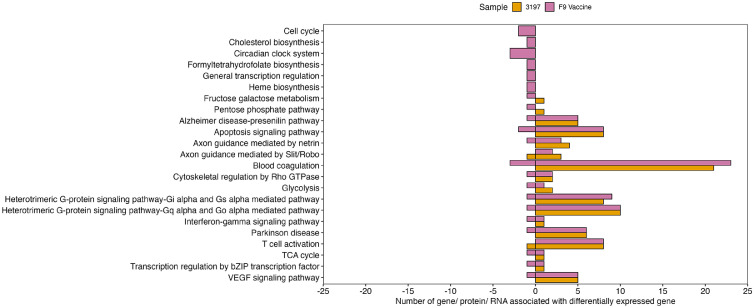
Differentially regulated host pathways in CRFK cells infected with FCV field strain 3197 or F9 vaccine strain. The number of proteins, RNA or genes associated with the differentially expressed genes in each infection group is shown with the downregulated genes represented < 0 and the upregulated genes represented > 0.

Pathway analysis was performed separately for upregulated and downregulated host genes in both field and vaccine infection groups. Overall, the vaccine infection group showed more enriched host pathways than the field infection group, with 117 significantly dysregulated pathways compared to 111 pathways in the field infection group (Supplementary figure 1). All pathways dysregulated in the field infection group were dysregulated in the vaccine infection group. Twenty-three host pathways were differentially regulated between field and vaccine infection groups and are shown in Fig. 7. Of these, downregulated genes in the vaccine infection group uniquely dysregulated six pathways compared to the field infection group. In the field infection group, both upregulated and downregulated genes contributed to pathways involved in axon guidance mediated by Slit/Robo and T-cell activation, whereas in the vaccine infection group, only upregulated genes were involved in the differential regulation of these pathways. For the remaining thirteen differentially expressed pathways, upregulated genes were observed in both infection groups, while downregulated genes were observed in the vaccine infection group. Additionally, upregulated genes in the field infection group were associated with the regulation of the pentose phosphate pathway and fructose galactose metabolism, whereas in the vaccine infection group, these pathways contained downregulated genes.

### 3.7 Different host functions are enriched when exposed to field or vaccine FCV strains

**Fig. 8:**
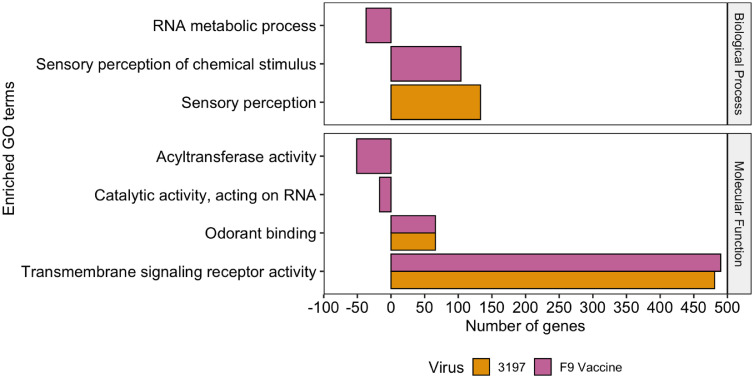
Statistical enriched GO terms of the cat genome from the up- and downregulated genes in the field and vaccine infection organised into two functional categories. The number of protein-encoding genes for each enriched GO term within each category is shown with the overrepresented genes represented > 0 and the underrepresented genes shown < 0.

Differentially expressed host genes of each infection group were analysed to determine their involvement in molecular function, biological processes and cellular components. In both field and vaccine infection groups, upregulated genes significantly contributed to odorant binding and transmembrane signalling receptors molecular functions, while in the vaccine strain, downregulated genes uniquely contributed to acyltransferase activity and catalytic activity, acting on RNA. Upregulated genes in the field infection group uniquely enriched sensory perception biological processes, whereas in the vaccine infection group, downregulated genes uniquely suppressed RNA metabolic processes, and upregulated genes uniquely enriched sensory perception of chemical stimulus biological processes (Fig. 8). No cellular components were significantly enriched (*P* < 0.05) among the upregulated or downregulated genes in either field or vaccine infection groups.

## 4 Discussion

FCV infections are associated with a range of clinical outcomes, which vary from mild URTD to virulent systemic disease (VSD). This variation may be associated with differences in viral strain virulence and host responses during infection. The difficulties in culturing human caliciviruses *in vitro* highlights the value of FCV cell culture models to explore viral pathogenesis, and host responses associated with viral strain virulence, in this family of viruses. In this study, infection of CRFK cells using either the F9 vaccine strain or the field strain 3197 showed differentially expressed host genes compared to uninfected cells. Both FCV infection strains induced the upregulation of genes encoding inflammatory mediators, stress response proteins and cell adhesion molecules, which is consistent with the activation of innate immune and cellular stress pathways following viral infection. Conversely, downregulated genes in both field and vaccine infection groups suppressed cell signalling, metabolism and extracellular matrix maintenance activity, which dysregulate host pathways involved in cellular homeostasis and survival. Differences in transcriptional profiles between field and vaccine strains showed unique gene expression associated with membrane signalling, vesicular tracking and cytoskeletal organisation. These findings suggest viral strain virulence may differentially influence cell signalling and injury responses. The vaccine infection group showed unique dysregulation of genes involved in chromatin regulation, cytokine signalling and mitochondrial metabolism. These findings indicate transcriptomes of CRFK cells infected with FCV strains can show host gene expression profiles associated with viral strain virulence which may contribute to variations in FCV infection outcome.

Several FCV strains co-circulate within catteries and shelters, and FCV shows high mutation rates in endemic cat populations (Lawson et al., 2019). Highly virulent VS-FCV strains are associated with mortality rates up to 50 % (Hurley and Sykes, 2003). The archived FCV field strain 3197 used in this study was originally collected from an 11 year old female Siamese cat that developed clinical signs consistent with VS-FCV, including progressive respiratory disease, oral ulceration, mandibular swelling, necrotising cellulitis and subcutaneous oedema (Stockley, 2013). Current FCV vaccines, including those based on the attenuated F9 strain first isolated in 1958, can reduce the severity of disease but do not prevent infection or reinfection with heterologous strains (Pedersen and Hawkins, 1995; Spiri et al., 2021). In this study, infecting CRFK cells with either the 3197 field strain or the F9 vaccine allowed comparison of transcriptional responses between viral strain virulence characteristics. While CRFK cells used in this study are fibroblastic kidney cells, which are not natural target cells of FCV, CRFK cells are widely used models for FCV propagation and pathogenesis studies (Spiri et al., 2021). Field and vaccine strains showed similar replication kinetics and induced CPE consistent with previously reported FCV-induced CPE in CRFK cells (Kim et al., 2010). Additionally, host transcriptional changes in FCV infected cells compared to uninfected cells showed similar dysregulated inflammatory responses associated with cytokines such as TNF-α and IL-10 to those observed in tissue samples of cats with VS-FCV compared to cats with mild or subclinical infections (Foley et al., 2006). These similarities in gene expression between *in vitro* and *in vivo* settings suggest FCV infections *in vitro* may show host-pathogen interactions that differ with viral strain virulence in a way that mirrors host responses observed in cats.

Following FCV infection, host genes encoding the pro-inflammatory cytokine TNF-α, the chemokine receptor IL-4R, and adhesion and stress molecules were upregulated in cells infected with FCV field or vaccine strain. In specific pathogen-free (SPF) cats vaccinated with a modified-live FCV strain, mRNA transcription of TNF-α and IL-6 were upregulated in the peripheral blood, alongside cytokines and proteins which regulate the differentiation of T cells (Al-Molawi et al., 2003; Natoni et al., 2006; Roberts et al., 2003; Sosnovtsev et al., 2003). Upon viral entry, pattern recognition receptors (PRRs) such as endosomal Toll-like receptors (TLRs) and cytoplasmic sensors recognise viral RNA can activate signalling cascades which results in the release of pro-inflammatory cytokines, chemokines and interferons (IFNs). The resulting type 1 IFN response, together with IL-12, promotes Th1 differentiation and IFN-γ production by cytotoxic T cells, while IL-4 primes Th2 differentiation and B cell activation to the site of infection (Swain et al., 2012). In this study, downregulated genes in the field infection group distinctly suppressed T cell activation pathways, while the vaccine strain distinctly upregulated IL-22 encoding genes and downregulated genes involved in IFN-γ signalling pathways. Past studies have shown FCV protease interactions with host transcription factors AP-1 and NF-kB can increase TNF-α mRNA expression, and the VPg protein can activate cyclooxygenase (COX) 2 expression through the MEK1-ERK1/2 pathway in kittens (Alhatlani et al., 2015; Thorne and Goodfellow, 2014). These findings highlight host gene expression observed during FCV infection *in vitro* can resemble host responses associated with innate immune activation and inflammatory responses observed *in vivo*.

Following viral entry, calicivirus genome translation begins immediately after the viral genome is released in the cytoplasm. Translation of the uncapped FCV genome relies on interactions between VPg and host translation initiation factors to recruit ribosomes and initiate viral replication (Sosnovtsev et al., 2002; Willcocks et al., 2004). In this study, the downregulation of genes involved in cellular metabolism, transcription regulation, cell signalling and extracellular matrix maintenance corresponded with visible CPE and dysregulated translational resources associated with cell death. Previous studies have shown that FCV activates several caspases involved in apoptosis and can downregulate anti-apoptotic proteins such as survivin and XIAP in CRFK cells (Geissler et al., 2002; Hofmann-Lehmann et al., 2022). These pro-apoptotic effects are attributed to the viral leader capsid (LC) protein, which can induce apoptosis and cytopathic changes to release mature virions for viral dissemination (Abente et al., 2013; Barrera-Vazquez et al., 2019). These findings suggest FCV can suppress pathways that maintain cell survival, metabolic balance and extracellular matrix integrity to induce apoptosis which may contribute to viral pathogenesis.

Viral transcripts of field and vaccine strains showed similar transcription patterns across the genome during early stages of viral replication. A notable peak in the field strain corresponded to region encoding the RNA-dependent RNA polymerase (RdRp) at the 3′ end of ORF1, while peaks in the vaccine strain corresponded to regions encoding viral proteins p30 and VPg. However, ORF1 of FCV encodes a single polyprotein that is processed by the viral protease into six NS proteins (NS1 through NS6/7). The protease, encoded in NS6, and the RdRp, encoded in NS7, are expressed as a single fusion protein that is not further cleaved (Penaflor-Tellez et al., 2019). This suggests the viral reads in this study likely represent a mixture of genomic and subgenomic RNA species. Therefore, investigation of the feline immune response and FCV pathogenesis is needed to better understand host responses associated with viral strain virulence and severe disease outcomes.

By comparing the host transcriptomes during infection with virulent and attenuated FCV strains, this study demonstrates CRFK cells infected FCV show differential host gene expression profiles associated with viral strain virulence. These findings highlight host-pathogen interactions during early stages of infection may contribute to FCV pathogenesis and variation in disease outcomes. Future studies using primary feline epithelial cells or organoid models that more closely mimic natural FCV infection sites will help validate the findings of this study and inform our understanding of feline immune responses to FCV.

## Funding

This research did not receive any specific grant from funding agencies in the public, commercial or not-for-profit sectors.

## CRediT authorship contribution statement

**Emily Kwan:** Writing – original draft, Visualization, Validation, Project administration, Methodology, Investigation, Formal analysis, Data curation. **Alistair R. Legione:** Writing – review & editing, Supervision, Resources, Conceptualization. **Carol A. Hartley:** Writing – review & editing, Supervision, Resources, Conceptualization. **Joanne M. Devlin:** Writing – review & editing, Supervision, Conceptualization.

## Declaration of competing interest

The authors declare that the research was conducted in the absence of any relationships that could be considered as potential conflict of interest.

## Data Availability

The datasets generated and/or analysed during the current study are available in the GenBank repository BioProject Accession No. PRJNA1082348.

## Appendix Supplementary materials

Image, application 1

Image, image 2
