# Supplementary material for: Transcriptomic analysis of Crandell-Rees feline kidney cell infections with field and vaccine feline calicivirus strains
Source: Virus Res. 2025 Dec 21;364:199681. doi: 10.1016/j.virusres.2025.199681 (PMC12818158; doi:10.1016/j.virusres.2025.199681)
Supplement: Supplementary file 1 [file mmc1.docx]

**Supplementary material**

**Supplementary table 1.** Average viral titre of field and vaccine FCV strains in CRFK cells at six timepoints over 24 hours. ^1^

| Timepoint (h.p.i.) | **FCV strain** | | | | | | | |
| --- | --- | --- | --- | --- | --- | --- | --- | --- |
|  | 3197 | 3198 | 3199 | 3200 | 3201 | 86/68 | FCV14 | F9 Vaccine |
| 1 | 3.80 ± 0.50^AC^ | 3.40 ± 0.17^A^ | 3.60 ± 0.00^A^ | 3.40 ± 0.17^A^ | 3.33 ± 0.40^AC^ | 5.57 ± 0.25^B^ | 4.00 ± 0.17^C^ | 3.73 ± 0.60^AC^ |
| 3 | 3.67 ± 0.12^A^ | 3.33 ± 0.25^AB^ | 3.77 ± 0.29^AD^ | 3.40 ± 0.53^ABD^ | 3.07 ± 0.25^B^ | 5.43 ± 0.29^C^ | 3.73 ± 0.12^A^ | 4.17 ± 0.12^D^ |
| 9 | 9.73 ± 0.12^A^ | 8.00 ± 0.79^B^ | 8.33 ± 0.40^B^ | 8.10 ± 0.00^B^ | 8.40 ± 0.36^B^ | 11.63 ± 0.29^C^ | 10.40 ± 0.79^AC^ | 8.73 ± 0.17^A^ |
| 12 | 10.00 ± 0.17^AD^ | 8.60 ± 0.00^B^ | 9.73 ± 0.12^AB^ | 9.17 ± 0.40^B^ | 9.67 ± 0.40^AB^ | 10.67 ± 0.51^D^ | 9.83 ± 0.40^CDE^ | 9.50 ± 0.12^C^ |
| 18 | 11.30 ± 0.87^A^ | 9.83 ± 0.87^AB^ | 10.07 ± 0.46^AB^ | 9.40 ± 0.53^B^ | 9.40 ± 0.17^B^ | 10.17 ± 0.51^AB^ | 11.07 ± 0.46^A^ | 10.73 ± 0.81^AB^ |
| 24 | 11.60 ± 0.69^AD^ | 10.67 ± 1.10^ABCD^ | 11.33 ± 0.46^AD^ | 9.73 ± 0.98^ABC^ | 9.10 ± 1.00^B^ | 9.30 ± 0.87^BC^ | 12.07 ± 0.46^D^ | 7.50 ± 0.85^BE^ |

^1^Statistical analysis was performed using Student's t-test and P- values < 0.05 considered significant. Values with the same superscript letter at each timepoint are not statistically different.

**Supplementary figure 1**. Total host pathways in CRFK cells infected with FCV field strain 3197 or F9 vaccine strain. The number of proteins, RNA or genes associated with the differentially expressed genes in each infection group is shown with the upregulated genes represented > 0 and downregulated genes indicated < 0.
