# Supplementary figures and images for: Transcriptomic analysis of Crandell-Rees feline kidney cell infections with field and vaccine feline calicivirus strains
Source: Virus Res. 2025 Dec 21;364:199681. doi: 10.1016/j.virusres.2025.199681 (PMC12818158; doi:10.1016/j.virusres.2025.199681)

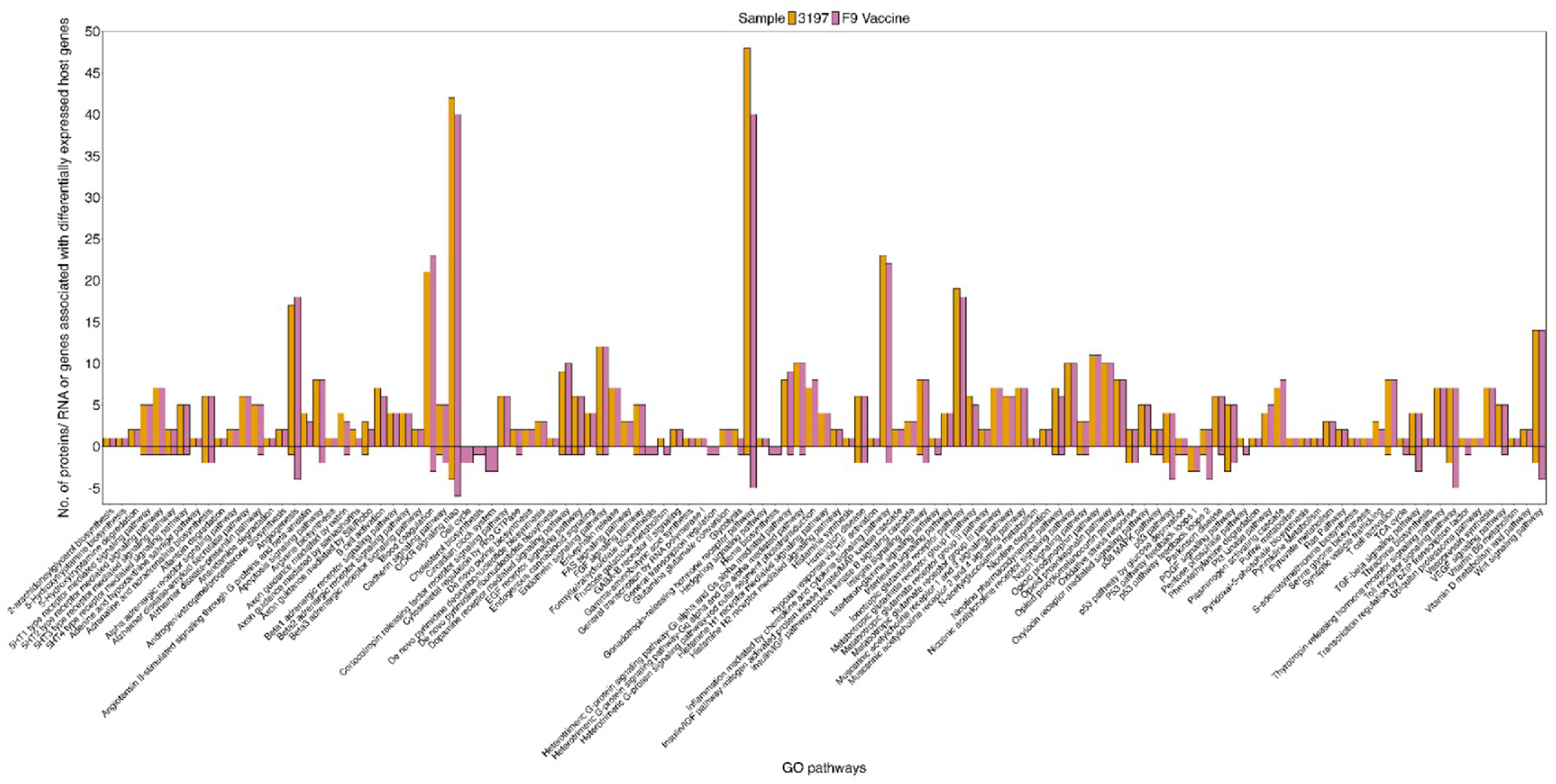

Supplement: Supplementary file 2 [file mmc2.jpg]
